# AXL knockdown gene signature reveals a drug repurposing opportunity for a class of antipsychotics to reduce growth and metastasis of triple-negative breast cancer

**DOI:** 10.18632/oncotarget.26725

**Published:** 2019-03-12

**Authors:** Marie-Anne Goyette, Rebecca Cusseddu, Islam Elkholi, Afnan Abu-Thuraia, Nehme El-Hachem, Benjamin Haibe-Kains, Jean-Philippe Gratton, Jean-François Côté

**Affiliations:** ^1^ Montreal Clinical Research Institute (IRCM), Montréal, QC, H2W 1R7, Canada; ^2^ Molecular Biology Programs, Université de Montréal, Montréal, QC, H3T 1J4, Canada; ^3^ Princess Margaret Cancer Centre, Toronto, University Health Network, ON M5G 1L7, Canada; ^4^ Department of Medical Biophysics, University of Toronto, Toronto, ON M5G 1L7, Canada; ^5^ Department of Computer Science, University of Toronto, Toronto, ON M5T 3A1, Canada; ^6^ Ontario Institute for Cancer Research, Toronto, ON M5G 1L7, Canada; ^7^ Vector Institute, Toronto, ON M5G 1L7, Canada; ^8^ Department of Pharmacology and Physiology, Université de Montréal, Montréal, QC, H3C 3J7, Canada; ^9^ Department of Biochemistry and Molecular Medicine, Université de Montréal, Montréal, QC, H3C 3J7, Canada; ^10^ Department of Anatomy and Cell Biology, McGill University, Montréal, QC, H3A 0C7, Canada

**Keywords:** triple-negative breast cancer, drug repurposing, AXL, phenothiazines, metastasis

## Abstract

Triple-Negative Breast Cancer (TNBC) is an aggressive cancer subtype that is associated with a poor prognosis due to its propensity to form metastases. The receptor tyrosine kinase AXL plays a role in tumor cell dissemination and its expression in breast cancers correlates with poor patient survival. Here, we explored whether already used drugs might elicit a gene signature similar to that seen with AXL knockdown in TNBC cells and which could, therefore, offer an opportunity for drug repurposing. To this end, we queried the Connectivity Map with an AXL gene signature which revealed a class of dopamine receptors antagonists named phenothiazines (Thioridazine, Fluphenazine and Trifluoperazine) typically used as anti-psychotics. We next tested if these drugs, similarly to AXL depletion, were able to limit growth and metastatic progression of TNBC cells and found that phenothiazines are able to reduce cell invasion, proliferation, viability and increase apoptosis of TNBC cells *in vitro*. Mechanistically, these drugs did not affect AXL activity but instead reduced PI3K/AKT/mTOR and ERK signaling. When administered to mice bearing TNBC xenografts, phenothiazines were able to reduce tumor growth and metastatic burden. Collectively, these results suggest that these antipsychotics display anti-tumor and anti-metastatic activity and that they could potentially be repurposed, in combination with standard chemotherapy, for the treatment of TNBC.

## INTRODUCTION

Triple-Negative Breast Cancer (TNBC) is an aggressive molecular subtype that is usually associated with increased metastatic incidences and a poor prognosis [1–3]. This particularly aggressive cancer subtype represents 10–20% of breast cancer cases and is characterized by the low expression of estrogen receptor (ER), progesterone receptor (PR) and HER2 receptor. Currently, no efficient targeted therapies are available to treat this aggressive cancer. Being routinely treated with standard chemotherapies, TNBC patients are commonly subject to serious side effects and are at high risk of developing drug resistance, tumor relapses and metastases [4]. Therefore, developing effective treatments to treat TNBC represents one of the most pressing challenge in breast cancer research.

AXL is a member of the TAM family of receptor tyrosine kinases (RTKs) that also includes TYRO3 and MER. These RTKs have well-established roles in various steps of tumorigenesis including proliferation, survival, migration, angiogenesis, immune evasion and drug resistance [5]. In breast cancer cell lines, AXL expression is restricted to TNBC cells that display strong mesenchymal phenotypes [6]. Functionally, AXL-driven mesenchymal characteristics endow tumor cells with an increased invasive phenotype and a resistance to chemotherapeutics [6–9]. Mechanistically, AXL promotes tumorigenesis and metastasis in part by activating key signaling molecules, including PI3K/AKT, MAPKs and Epithelial-to-Mesenchymal Transition (EMT) modulators [5, 10]. Indeed, we and others have shown that the expression of AXL in human solid tumors is linked to a poor prognosis and is essential for metastasis in breast cancer models of TNBC and HER2+ subtypes *in vivo* [8, 10]. A significant body of work, therefore, has established AXL as a promising clinical target for managing multiple cancers, and TNBC in particular. Consequently, a small molecule inhibitor specific to AXL (R428; also known as BGB-324 or Bemcentinib) is currently under investigation in a phase II clinical trial for various cancers, including non-operable and metastatic TNBC [11, 12]. While this specific AXL inhibitor may soon reach the clinic and is promising in terms of overall survival and response rate as suggested by data from preclinical models [13], unfavorable outcomes including problems with drug tolerability and resistance could also arise. In this case, novel alternative approaches mimicking AXL inhibition might be of importance for advanced TNBC patient care.

Drug repurposing involves the identification of novel clinical applications of previously approved drugs. Since these drugs are approved by the FDA or other regulatory agencies and are used in the clinic, their safety, toxicity and pharmacological properties have already been thoroughly characterized. Consequently, drug repurposing represents a cost- and time-effective approach to identify novel pharmacotherapies to manage aggressive conditions such as TNBC. A powerful tool to identify drugs for repurposing is the use of large collections of genome-wide transcriptional gene expression datasets from human cells treated with a variety of FDA approved and experimental small molecules.

In this study, using the novel integrative package for pharmacogenomics PharmacoGx [14–16], we found that the phenothiazine class of antipsychotics (Thioridazine (THZ), Fluphenazine (FLZ) and Trifluoperazine (TFP)) displays a gene signature similar to that seen with *AXL* depletion in TNBC cells. *In vitro*, phenothiazines were able to reduce cell invasion, proliferation, tumorsphere formation and increase cell death in TNBC cells and these actions correlated with reduced signalling from PI3K/AKT/mTOR and ERK pathways. *In vivo*, the administration of phenothiazines to mice bearing xenografts of MDA-MB-231 TNBC cells reduced tumor growth and metastatic burden. These findings identify members of this class of drugs as potential candidates for repurposing in TNBC.

## RESULTS

### Pharmacogenomics identifies phenothiazines, a class of antipsychotics, as candidates for drug repurposing in TNBC

TNBC is currently treated by chemotherapy and carries a risk of drug resistance, relapse and metastasis [3]. Because AXL is emerging as a promising drug target to limit certain metastatic cancers including TNBC [5, 8, 10], we sought to identify drugs that could be repurposed based on their induction of a gene signature similar to that seen with AXL depletion. The aggressive AXL^high^ TNBC cell line MDA-MB-231 was subjected to siRNA-mediated knockdown of *AXL* and RNA-seq was performed to generate an *AXL* gene signature (GSE120268). To validate this signature, we first performed Gene Ontology and Gene Set Enrichment Analysis (GSEA) to assess enrichment of biological processes and pathways [17]. Many of the genes associated with known physiological roles of AXL including proliferation, migration and regulation of EMT, were found to be modulated by AXL depletion (Supplementary Figure 1A, 1C). Furthermore, different pathways related to AXL were enriched including PI3K/AKT, mTOR and MAPK signalling pathways (Supplementary Figure 1B–1C). Altogether, these results suggest that the generated *AXL* gene signature is representative of AXL depletion in cancer cells and is a valid tool to interrogate pharmacogenomics databases.

**Figure 1:**
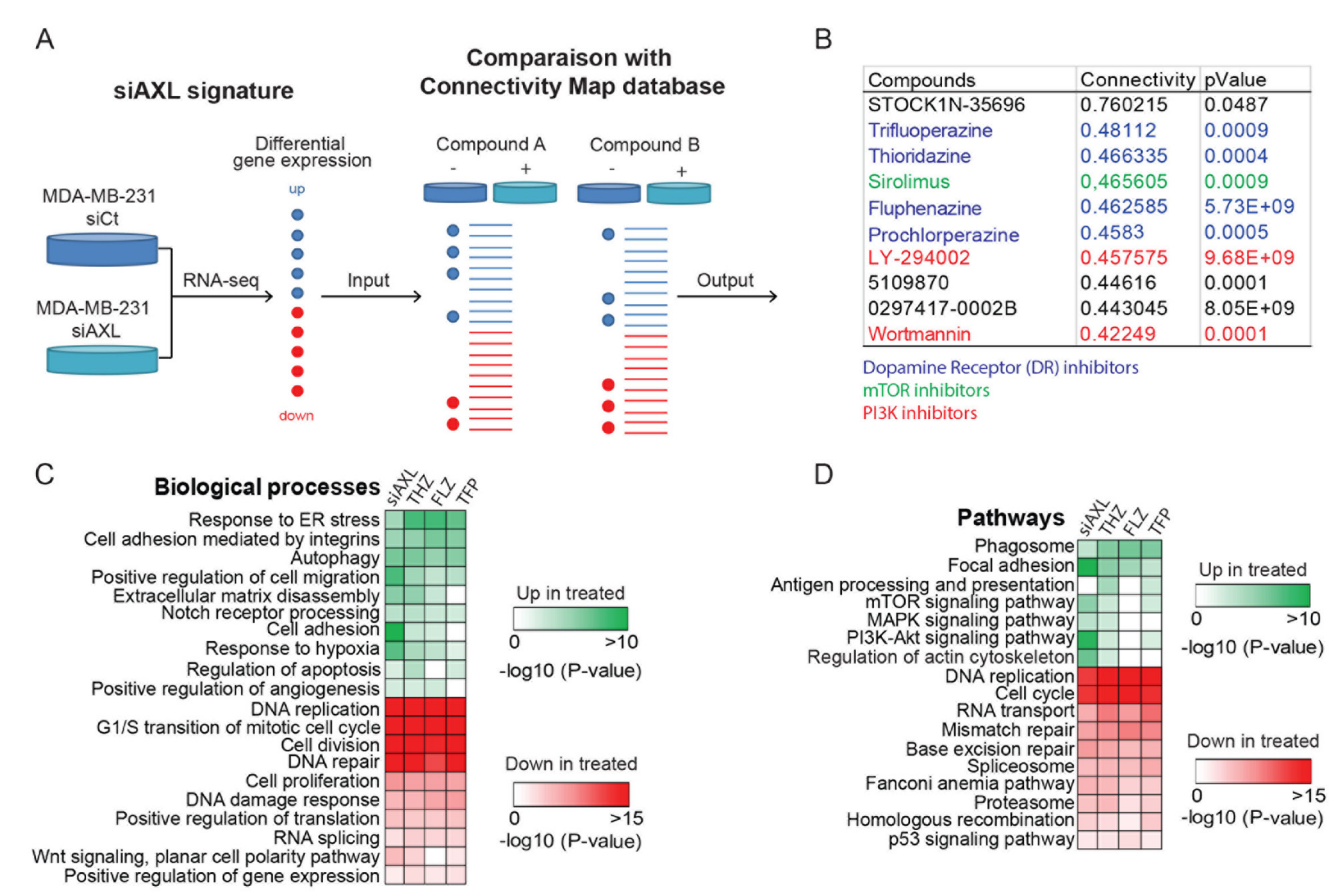
AXL knockdown gene expression signature in MDA-MB-231 is similar to the signature of a class of antipsychotics (**A**) Representation of the Connectivity map (CMap) approach used to define FDA- approved drugs that resemble AXL depletion in the MDA-MB-231 TNBC cell line. MDA-MB-231 cells treated with siCtl or siAXL were used to generate a gene expression signature that was compared to the CMap reference database to identify compounds that produce similar gene expression modulations. (**B**) Table of the top 10 hits with the best connectivity with the AXL knockdown signature. This CMap analysis includes a class of dopamine receptor inhibitors, the phenothiazines that were selected for further characterization. Other families of inhibitors are also highlighted. (**C**–**D**) The gene signatures changes induced by phenothiazines treatments were extracted from PharmacoGx and compared to our AXL knockdown signature. Gene Ontology analyses of the common modulated genes show the pathways and biological processes modulated by these treatments.

We next interrogated the Connectivity map (CMap), a database intersecting pharmacological drugs and genomics data, using our Bioconductor platform PharmacoGx to find known drugs that induce a response that mimics the *AXL* signature (Figure 1A) [14–16]. Approximately 50 compounds were identified (*P*-value < 0.05, Supplementary Table 1) which we reduced to the 10 compounds with the highest positive connectivity score (Figure 1B). Among the top hits, we found inhibitors of PI3K and mTOR whose pathways are known to be modulated downstream of AXL [5, 18] therefore validating our approach. The compound with the highest connectivity, STOCK1N-35696, is not well characterized and not currently used in the clinic. As such, it was not further investigated as part of our search for previously accepted drugs. Interestingly, four dopamine receptor (DR) inhibitors, belonging to the antipsychotic family of phenothiazines, were among the top hits in our screen (Figure 1B) [19]. Some members of this family of antipsychotics were recently reported to have effects on proliferation, apoptosis, stemness and migration in several cancer types including melanoma [20, 21], ovarian cancer [22, 23], breast cancer [24, 25], cervical and endometrial cancer [26] and lung cancer [27, 28]. Indeed, previous work has shown that DRs are enriched in leukemic and primary TNBC cancer stem-like cells, suggesting that THZ could act through these receptors to limit their expansion [29]. However, our RNA-seq data suggest that DRs are minimally expressed in MDA-MB-231 cells, implying that their mechanism of action is likely to be different in these cells. THZ and TFP have also been linked to a decrease in activity of PI3K/AKT pathway, however, the mechanism in this context remains unexplored [22, 23, 26]. In this study, we selected Thioridazine (THZ), Fluphenazine (FLZ) and Trifluoperazine (TFP) to further investigate their action on TNBC.

We next used PharmacoGx to extract the gene signatures of THZ, FLZ or TFP from CMap and identify the common gene expression patterns shared by cells when treated with these antipsychotics and compared these to the patterns obtained when AXL is depleted in MDA-MB-231 cells. By comparing Gene Ontology analyses across all treatments, we found that genes involved in the biological processes of cell migration, cell proliferation and apoptosis were modulated in all of the conditions (Figure 1C). Furthermore, genes involved in mTOR, MAPK and PI3K/AKT signaling pathways were also modulated, suggesting a possible mechanism of action for these drugs (Figure 1D). To further confirm the effect of these compounds in the context of TNBC, we also performed RNA sequencing of MDA-MB-231 treated with one of the phenothiazines, THZ, considering that all three drugs have a similar signature in CMap. Accordingly, we found that the pattern of biological processes and pathways modulated by THZ treatment to be similar to those found using the PharmacoGx datasets (Supplementary Figure 1D). Thus, these analyses suggest that these antipsychotics could be candidates for drug repurposing in TNBC.

### Phenothiazine-family antipsychotics reduce migration and invasion of TNBC cells

**Figure 2:**
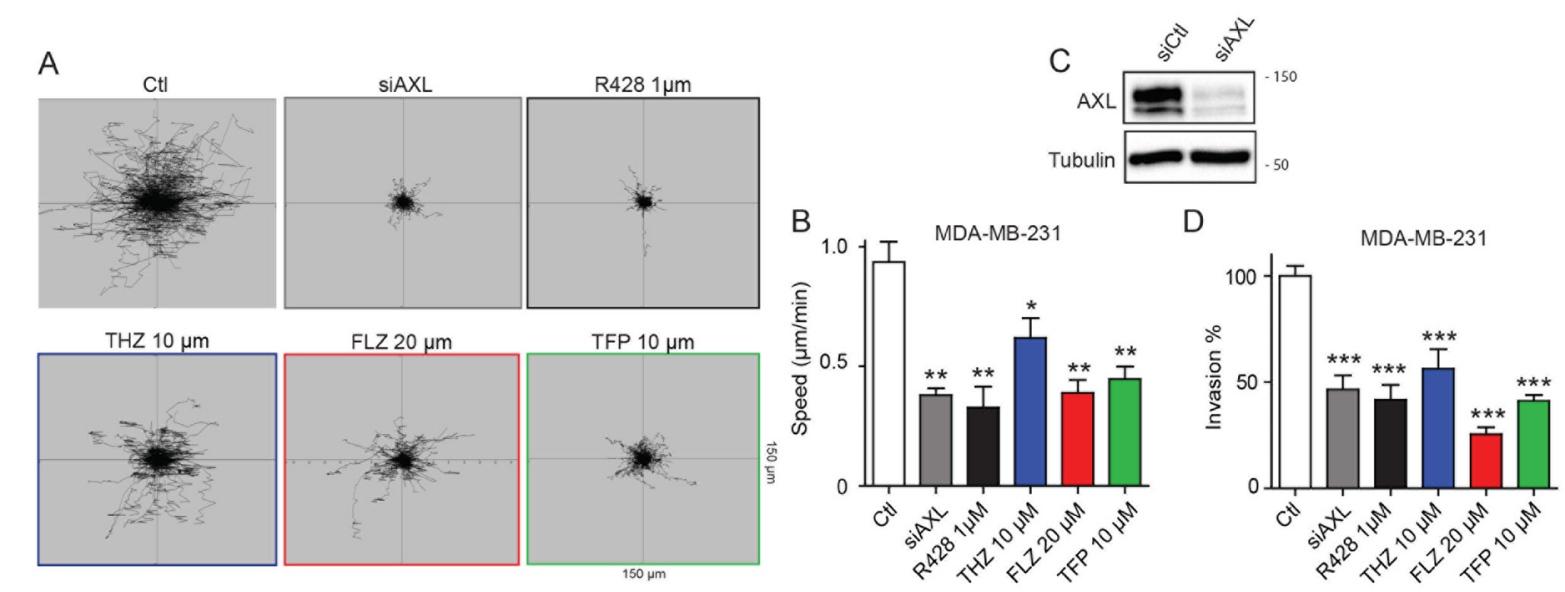
Treatments with THZ, FLZ and TFP reduces the migration potential of TNBC cells (**A**, **B**) Treatments with the indicated concentrations of phenothiazines reduce cell migration speed. The migration of MDA-MB-231 cells was tracked by time-lapse microscopy over a 6 h period in the presence of phenothiazines or AXL inhibitor or siRNA as positive controls. (^**^p = 0.0029, ^**^p = 0.0044, ^*^p = 0.0365, ^**^p = 0.0016) (*n* = 3). Data are represented as mean ± SEM. (**C**) Cells were transfected with the indicated siRNA and knockdown of AXL was validated by Western Blot. Equal loading of proteins between samples was confirmed by blotting against Tubulin. (**D**) Inhibition of AXL via siRNA and the small molecule inhibitor R428 or treatments with the antipsychotics reduce invasion of MDA-MB-231 cells in a Boyden invasion assay towards serum as an attractant (^***^p < 0.0001). (*n* = 3) Data are represented as mean ± SEM.

Since AXL is a driver of cell migration and invasion in TNBC cells [8], we investigated whether phenothiazines can interfere with these processes. As expected, targeting AXL either pharmacologically (using the small molecule inhibitor R428) or genetically through siRNA in MDA-MB-231 cells decreased cell migration speed as measured by live cell imaging (Figure 2A–2C, Supplementary Figure 2C, Supplementary Movie 1). Treatment of MDA-MB-231 cells with the phenothiazines similarly reduced cell migration (Figure 2A–2C, Supplementary Figure 2C, Supplementary Movie 1). Similar attenuation in cell migration was also observed when the TNBC cell line Hs578T was treated with THZ, FLZ or TFP (Supplementary Figure 2A). Similar to AXL pharmacological inhibition or siRNA-mediated depletion, treatment of MDA-MB-231 cells with phenothiazines also reduced cell invasion in a matrigel Boyden invasion assay towards serum (Figure 2D). However, only the treatment of Hs578T cells with FLZ and TFP, but not THZ, was able to decrease their invasion (Supplementary Figure 2B). Collectively, these results suggest that certain phenothiazine antipsychotics can reduce the motility and invasiveness of TNBC cells.

### Phenothiazines reduce the proliferation of TNBC cells

**Figure 3:**
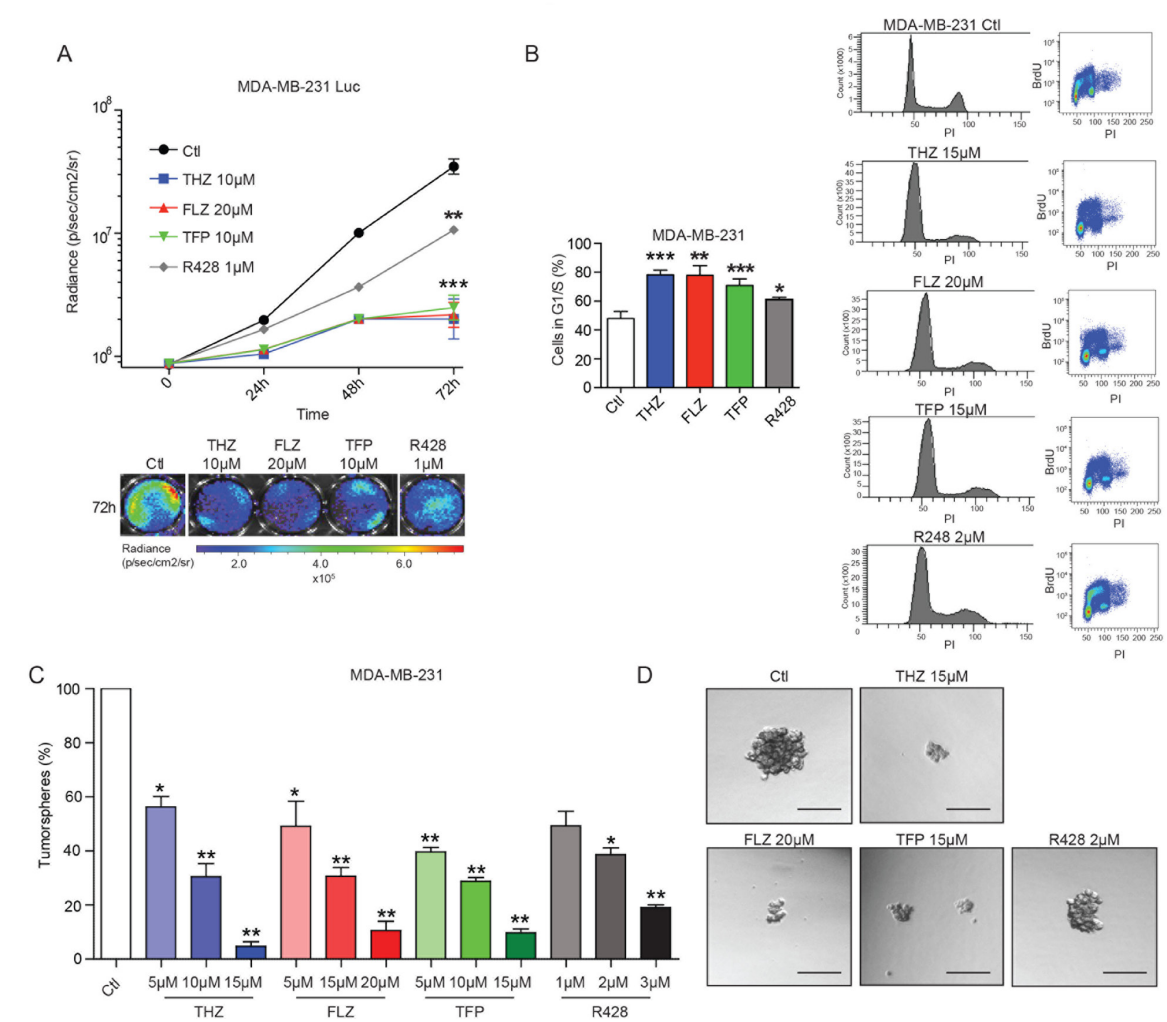
The phenothiazines reduce cell proliferation and induce cell cycle arrest of TNBC cells (**A**) The antipsychotics and AXL inhibitor R428 reduce the proliferation of the MDA-MB-231-Luc cells as shown by quantification of the luminescence signal (expressed in radiance) over a 72 h period (^***^p = 0.0002, *p* = 0.0009, ^**^p = 0.0041) (*n* = 4). (**B**) Treatments with the antipsychotics and R428 led to an increase in the number of cells in G1/S. Cell cycle analyses were done using FACS analyses of BrdU-stained MDA-MB-231 cells. (^***^p = 0.0009, ^**^p = 0.0033, ^**^p = 0.0041, ^*^p = 0.038) (*n* = 3). Data are represented as mean ± SEM. (**C**–**D**) Tumorspheres formation of MDA-MB-231 cells is inhibited by THZ, FLZ, TFP and R428 in a dose-dependent manner (^*^p = 0.0375, ^**^p = 0.0088, ^**^p = 0.0018, ^*^p = 0.0279, ^**^p = 0.0057, ^**^p = 0.0023, ^**^p = 0.0096, ^**^p = 0.0054, ^**^p = 0.0022, ^*^p = 0.0126, ^**^p = 0.0033) (*n* = 3). Scale bar, 150 μm. Data are represented as mean ± SEM.

Further analysis of the RNA-Seq data revealed a shared effect of both AXL depletion and phenothiazine treatment on genes involved in cell proliferation, cell cycle and G1/S transition of the mitotic cell cycle (Figure 1C–1D, Supplementary Figure 3A). This prompted us to investigate whether phenothiazines might display anti-proliferative effect on TNBC cells. To test this, we used MDA-MB-231 cells engineered to express Luciferase (MDA-MB-231-Luc) and measured bioluminescence as a surrogate to quantify the number of cells at different time points of the treatment. We found that MDA-MB-231-Luc cells treated with either the AXL inhibitor R428 or phenothiazines displayed reduced proliferation in a dose-dependent manner (Figure 3A, Supplementary Figure 3B). Furthermore, BrdU flow cytometry analyses showed that both MDA-MB-231 and Hs578T cells treated with phenothiazines accumulate in G1/S (Figure 3B, Supplementary Figure 3C–3D). AXL inhibition with R428 led to the accumulation of cells in G1/S in MDA-MB-231 cells but this effect was not significant in Hs578T cells (Figure 3B, Supplementary Figure 3C–3D).

We next tested whether phenothiazines can decrease TNBC progression in a more direct way. The ability of forming tumorspheres and to proliferate in suspension in an *in vitro* context is suggested to be mediated by a subpopulation of cells with stem cell-like traits [30, 31]. Being capable of self-renewal in addition to giving rise to a progeny of cancer cells, this subpopulation of cells is also believed to maintain tumor progression *in vivo* [30, 31]. As such, we tested the effect of the AXL inhibitor R428 or the phenothiazines on tumorsphere formation and found that treating either MDA-MB-231 or Hs578T TNBC cells with R428, THZ, FLZ or TFP led to a dose-dependent decrease in tumorsphere formation (Figure 3C–3D, Supplementary Figure 3E–3F). Together, these results demonstrate that phenothiazines have anti-proliferative effects and also suggest a potential role for these drugs in decreasing the progression of TNBC.

### Phenothiazines cooperate with paclitaxel to reduce tumorsphere formation

**Figure 4:**
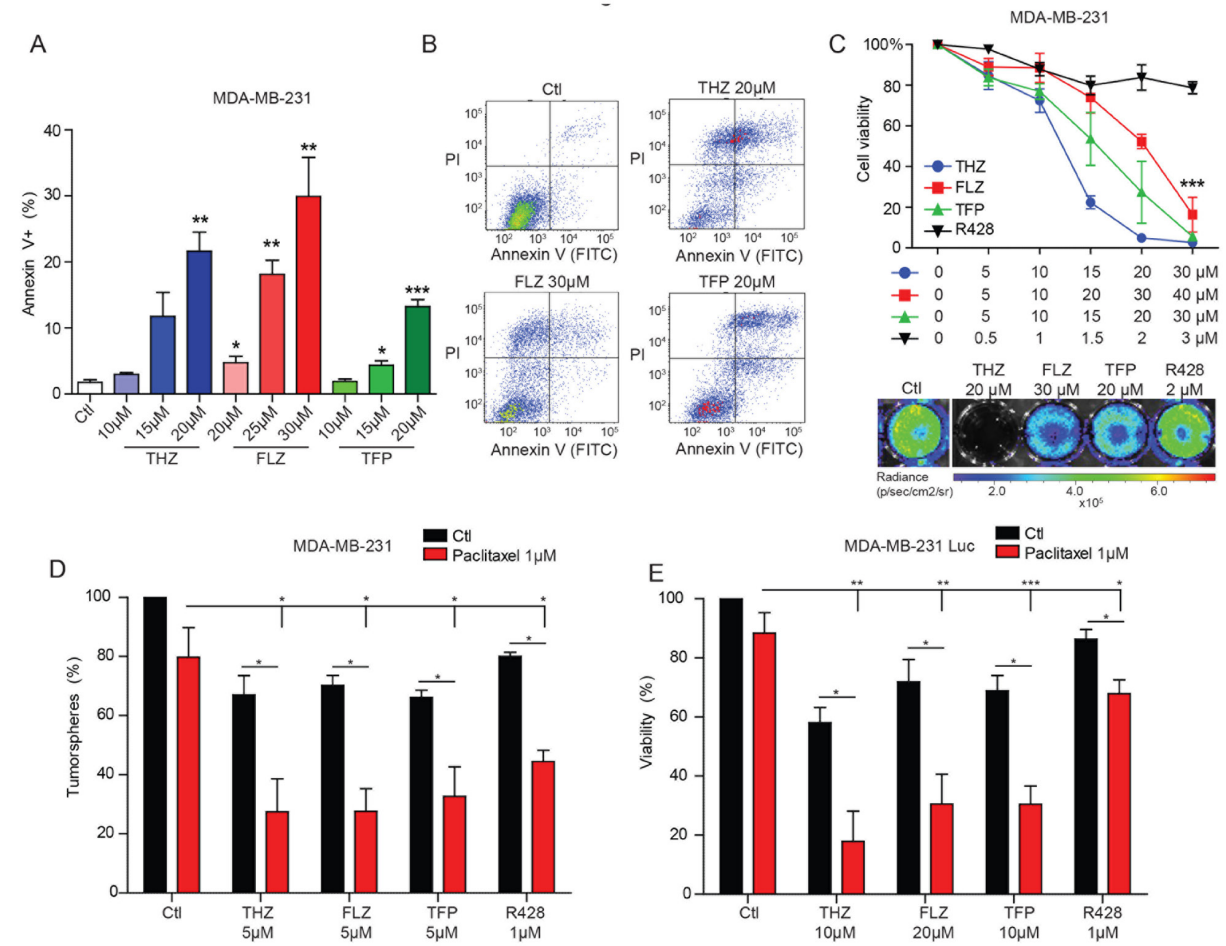
THZ, FLZ and TFP exhibit cytotoxic effects on TNBC cells (**A**, **B**) 24 h treatments with the antipsychotics induce apoptosis as evaluated by Annexin V and PI FACS analyses. (^**^p = 0.0024, ^*^p = 0.0473, ^**^p = 0.0017, ^**^p = 0.0089, ^*^p = 0.0369, ^***^p = 0.0006). (*n* = 3) Data are represented as mean ± SEM. (**C**) Phenothiazines reduce cell viability of MDA-MB-231-Luc cells as assessed by quantifying the bioluminescence signal after 24 h of treatment as a surrogate of cell number (^***^p < 0.0001). (*n* = 3) Data are represented as mean ± SEM. (**D**, **E**) The combination of paclitaxel and the antipsychotics or R428 increases the inhibitory effect of these drugs on tumorspheres formation and cell viability (^*^p = 0.0251, ^*^p = 0.0143, ^*^p = 0.0292, ^*^p = 0.0298/^*^p = 0.0187, ^*^p = 0.0209, ^*^p = 0.0294, ^**^p = 0.0034, ^**^p = 0.0014, ^**^p = 0.0033, ^***^p = 0.0008, ^*^p = 0.0466). (*n* = 3) Data are represented as mean ± SEM.

RNA-seq analyses also revealed a potential role for these compounds in apoptosis (Figure 1C). Indeed, we found that higher doses of phenothiazines promoted apoptosis and cell death, as determined by Annexin V and Propidium Iodide (PI) FACS analyses in MDA-MB-231 and Hs578T cells (Figure 4A–B, Supplementary Figure 4A–4B). In contrast, pharmacological inhibition of AXL with R428 or AXL depletion by siRNA did not induce apoptosis in either the MDA-MB-231 or the Hs578T cells, suggesting that broader, pleotropic effects may result from phenothiazine treatment in comparison to interfering with AXL function (Supplementary Figure 4C–4E). In agreement with these observations, viability of MDA-MB-231 cells was reduced by THZ, FLZ and TFP, but not by R428 treatment (Figure 4C).

Based on the observed anti-proliferative and pro-apoptotic effects of the phenothiazines, we asked whether these drugs might sensitize TNBC cells to the conventional chemotherapy treatment currently used to treat this form of breast cancer in the clinic [1, 4]. We tested whether the combination of the phenothiazines or R428 might cooperate with the chemotherapeutic paclitaxel to decrease tumorsphere formation and cell viability. While treatment with a suboptimal dose of paclitaxel alone did not show a significant effect, the combination with phenothiazines or R428 severely impaired tumorsphere formation and cell viability (Figure 4D–4E, Supplementary Figure 4F). These findings suggest that phenothiazines may warrant testing in the clinic as a means to sensitize the tumor cells to standard chemotherapy.

### THZ, FLZ and TFP affect the PI3K/AKT/mTOR and MAPK signalling pathways in TNBC

Phenothiazines are known to target dopamine receptors [19], yet our transcriptomics analyses revealed that MDA-MB-231 cells do not express significant amounts of the dopamine receptor family (Supplementary Figure 5A). We therefore investigated the mechanism of action of phenothiazines in TNBC cells. Since the *AXL* depletion gene signature was similar to that induced by phenothiazine treatment, we hypothesized that these drugs might be inhibiting AXL directly. To test this, we measured AXL phosphorylation levels (as a readout of its activation) upon treatment of MDA-MB-231 or Hs578T cells with the phenothiazines and found that it was unaffected, implying that these antipsychotics do not directly target AXL (Supplementary Figure 5B–5C).

Because not all of the characterized effects of phenothiazines on cell viability are reproduced with AXL inhibition (Figure 4C, Supplementary Figure 4C–4D), we reasoned that these treatments might have both overlapping and non-overlapping effects. This led us to test whether co-treatment of TNBC cells with R428 together with phenothiazines might result in an additive or synergistic effect on cell invasion or survival. Indeed, we found that the combination of R428 with the antipsychotics had a greater effect on cell invasion and viability than either treatment alone (Supplementary Figure 5D–5E).

**Figure 5:**
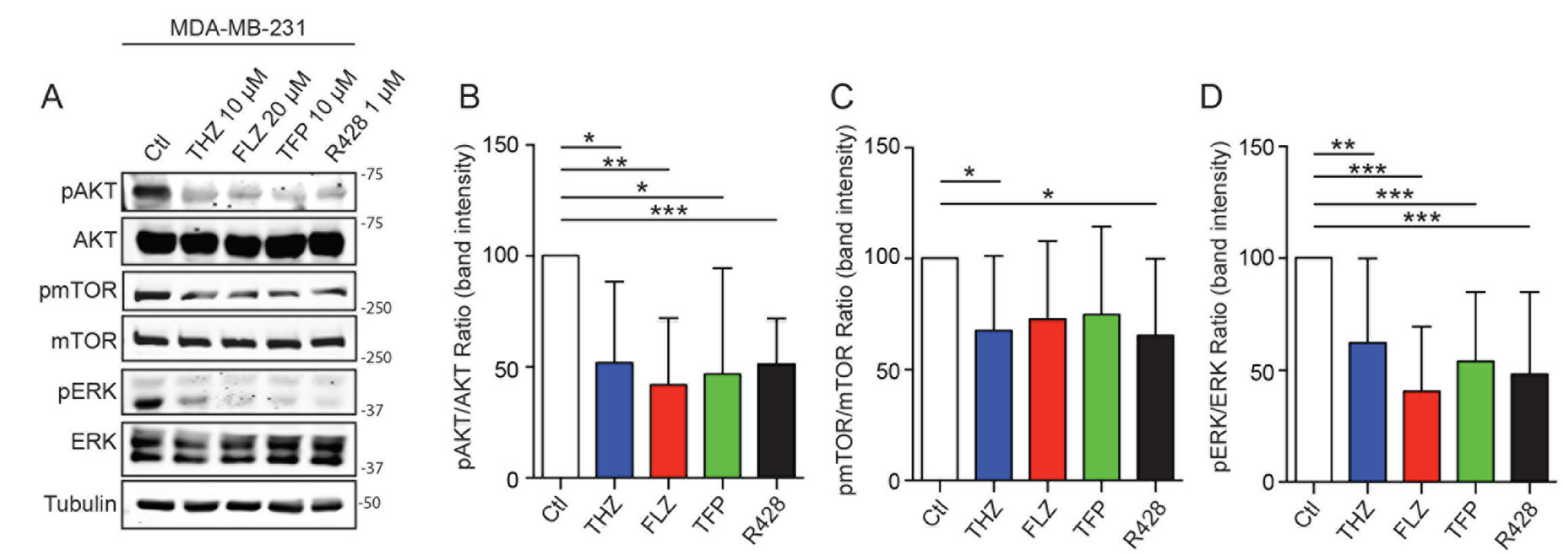
Phenothiazines treatments on TNBC affects PI3K/AKT/mTOR and MAPK pathways (**A**) THZ, FLZ, TFP and R428 reduce the phosphorylation of mTOR, ATK and ERK in MDA-MB-231. (**B**–**D**) Quantification of the phosphorylation intensity was done by measuring a ratio of band intensity of the phosphorylation signal over the signal of the total protein and is presented relative to the control condition for each protein. (^*^p = 0.0188, ^**^p = 0.0025, ^*^p = 0.0377, ^***^p = 0.0008 / ^**^p = 0.0084, ^***^p < 0.0001, ^***^p = 0.0004, ^***^p = 0.0006 / ^*^p = 0.0400, ^*^p = 0.0342). (*n* = 6–8) Data are represented as mean ± SEM.

Examination of the RNA-Seq analyses suggested that one mechanism of action for the phenothiazines might be through the PI3K-AKT, mTOR and MAPK signaling pathways (Figure 1C). Notably, the PI3K/AKT pathway was previously shown to be modulated by these drugs in different cancer types [22, 23, 26]. In the context of TNBC cells, we found that the phosphorylation levels of AKT, mTOR and ERK were reduced upon treatment with phenothiazines in MDA-MB-231 cells (Figure 5A–5D). In Hs578T, we observed a similar effect of phenothiazines on the activation levels of mTOR and AKT. However, the effects of the drugs were less consistent on ERK activity (Supplementary Figure 5F–5I). Thus, phenothiazines dampen the signalling from PI3K/AKT/mTOR and, to some extent, MAPK pathways in TNBC cells.

### THZ, FLZ and TFP reduce tumor growth and metastasis *in vivo*

**Figure 6:**
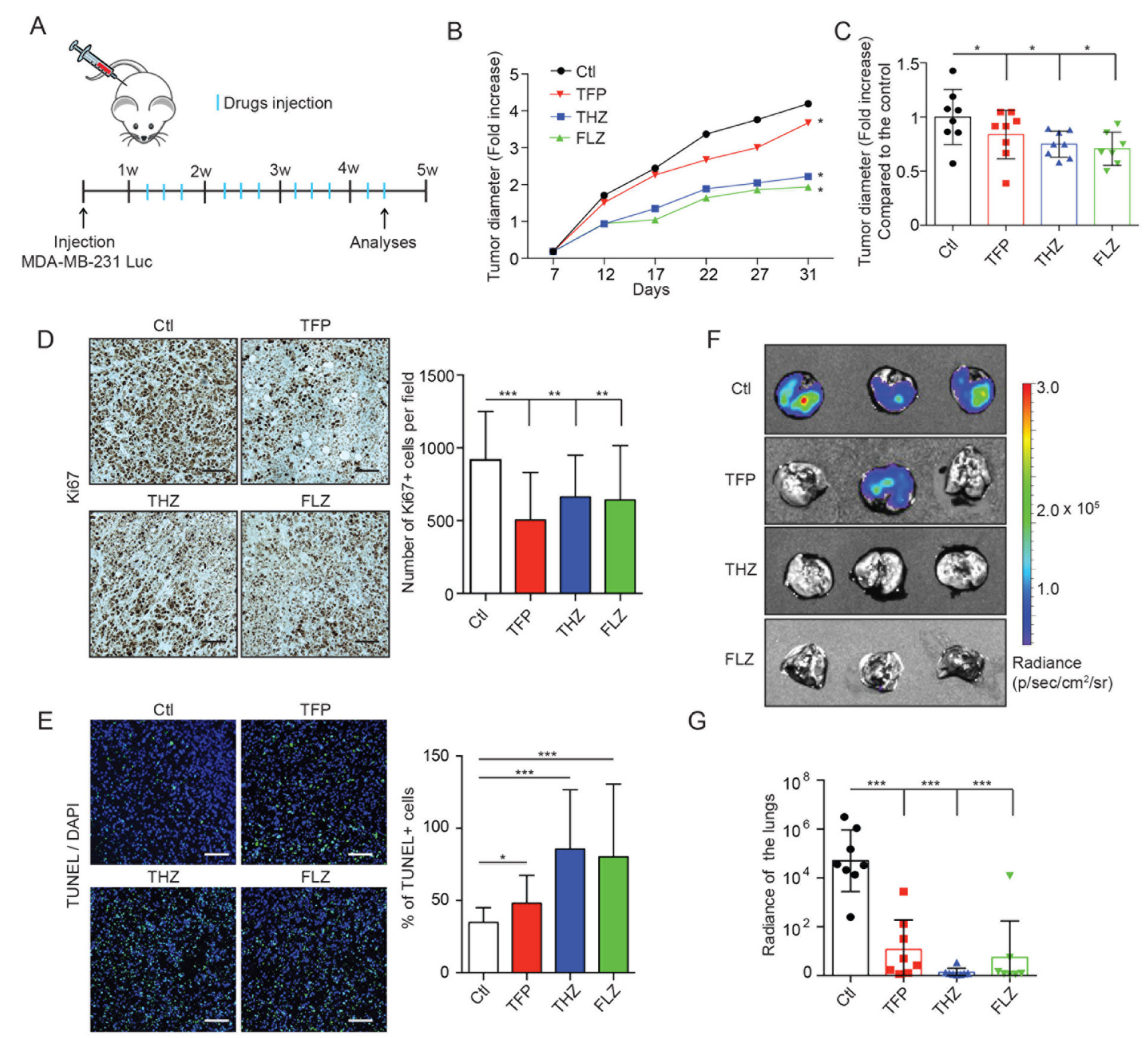
THZ, FLZ and TFP treatments reduce tumor growth and metastasis *in vivo* (**A**) Schematic showing the treatment of nude mice bearing mammary fat pad xenografts of MDA-MB-231-Luc cells. (**B**, **C**) Treatments of mice with MDA-MB-231-Luc mammary fat pad grafts with the antipsychotics reduce tumor growth. Tumor diameters were measured before the beginning of the treatments (7 days after the graft) to quantify the fold increase overtime. To make the growth curve, the tumor diameters were measured twice a week. (^*^p = 0.0242, ^*^p = 0.0197, ^*^p = 0.0344). (*n* = 8) Data are represented as mean ± SEM. (**D**) Tumors treated with THZ, FLZ and TFP showed reduced proliferation. Immunohistochemistry detection of Ki67 using DAB staining was done on primary tumors and Hematoxylin was used as a counterstain to reveal the nuclei. (^***^p < 0.0001, ^**^p = 0.0067, ^**^p = 0.0043) (*n* = 5) Scale bar: 50μm Data are represented as mean ± SEM. (**E**) Treatments with phenothiazines *in vivo* increased apoptosis shown by TUNEL fluorescent staining with DAPI as a counterstain for the nuclei. (^*^p = 0.0415, ^***^p < 0.0001, ^***^p = 0.0003) (*n* = 5) Scale bar: 50 μm Data are represented as mean ± SEM. (**F**, **G**) Treatments with THZ, FLZ and TFP reduce the metastatic progression of MDA-MB-231-Luc xenografts to the lungs. Representative images of lungs showing metastases. 31 days after tumor graft, at the end point of the growth experiments, mice were injected with Luciferin and lungs were dissected and imaged for bioluminescence signal to quantify the metastases. (^***^p < 0.0001). Data are represented as mean ± SEM.

To investigate the therapeutic value of these drugs *in vivo*, MDA-MB-231-Luc cells were grafted in the mammary fat pads of nude mice and tumors were allowed to grow for one week prior to treatment with the different phenothiazines as illustrated in Figure 6A. These experiments revealed that tumor growth, as measured by tumor diameter, was significantly impaired in treated mice compared to the untreated controls (Figure 6B–6C). Furthermore, cell proliferation, as assessed by Ki67 staining, was decreased (Figure 6D). Cell apoptosis, as assessed by a TUNEL assay, was increased in these tumors (Figure 6E). These results not only explained the observed *in vivo* phenotypes but also confirmed the *in vitro* effects observed in Figures 3–4.

Because 90% of cancer-related deaths are caused by metastasis and since AXL plays a major role in this process, we investigated whether phenothiazine treatment also impacted the metastatic progression of injected TNBC cells. The lungs of the mice, which are the preferred metastatic site of MDA-MB-231 cells in this spontaneous model of metastasis, were harvested at the experimental endpoint and were found by bioluminescence imaging to have a decreased metastatic burden as compared to untreated control animals (Figure 6F–6G). Furthermore, while the combination of the phenothiazines and paclitaxel *in vivo* tended to decrease the tumor growth and the metastatic burden, the difference between the single and the combined treatments were not statistically significant at the drug doses used (Supplementary Figure 6A–6B). Collectively, these results demonstrate that THZ, FLZ and TFP treatments can decrease tumor growth and the metastatic progression of TNBC cells *in vivo*, making these drugs a viable option for repurposing in the clinic.

## DISCUSSION

Managing TNBC represents such a major clinical challenge that novel therapeutic approaches are needed to significantly improve the survival and the quality of life of affected patients. Drug repurposing using pharmacogenomic approaches has emerged as a powerful resource to decrease the cost and time taken to translate novel treatment options from the bench to the bedside. In this study, we demonstrated that the gene expression signature resulting from *AXL* depletion can be used to identify potential drugs to repurpose against TNBC. Using the PharmacoGx platform, we were able to uncover members of a class of antipsychotics (THZ, FLZ and TFP) with the potential to antagonize TNBC progression. These compounds are typically described as dopamine receptor antagonists and have been used in the clinic to treat schizophrenia and other psychotic disorders [19]. Interestingly, some studies have demonstrated that schizophrenia patients have a low prevalence of melanoma that could be attributed to their treatment by neuroleptics, suggesting that these antipsychotic drugs might have a protective role against cancer [32, 33].

THZ was suggested to act on cancer cells via dopamine receptors that are expressed by a population of breast cancer and AML cells having stem cell-like properties [29]. Nonetheless, in our case, transcriptomic analyses revealed that MDA-MB-231 cells do not express significant amounts of the dopamine receptor family suggesting that these drugs may have different target(s) in TNBC cells. In agreement with our observations, a recent study investigated the effects of THZ on a panel of TNBC cells relative to their DRD2 expression [25]. While they found that THZ treatment of cells expressing DRD2 reduced their self-renewal and sphere formation via the DRD2/STAT3/IL-6 pathway, cell proliferation and survival were affected in a DRD2-independent manner in all cell lines via an unknown mechanism. Our study demonstrates that the effect of phenothiazines on proliferation and survival of TNBC cells expressing low levels of DRD2 could be mediated by decreasing the activity of the PI3K/AKT/mTOR and, to some extent, MAPK pathways. Interestingly, a similar pharmacogenomic approach that exploited a gene signature of AKT pathway inhibition identified the same three antipsychotics and suggested that at least THZ and TFP may be functioning through inhibition of the PI3K/AKT pathway [22, 23, 26]. While these findings suggest that phenothiazines may act directly on these signaling molecules, further work is needed to determine their exact targets in addition to DRD2.

The effects of phenothiazines seem to be cancer cell-dependent. Hs578T cells did not react in exactly the same manner as MDA-MB-231 cells and this is probably due to their different mutational profiles. The exact target(s) of phenothiazines in these cells should be determined to better understand the differences we and others have observed. Interestingly, we also determined that co-treating the TNBC cells with the AXL inhibitor R428 and phenothiazines further inhibited cell invasion and survival. These results suggest that there might be both common and unique effectors for these drugs. In fact, it is possible that the combination of these two drugs that individually affect PI3K/AKT signalling can further reduce cancer aggressiveness.

Members of the phenothiazine class of drugs have been shown to decrease proliferation and survival of different type of cancer cells [20, 22, 23, 25–27]. In this study, we report that THZ, FLZ and TFP have a negative impact on cell migration and invasion, proliferation and viability *in vitro* using TNBC cell lines as models. We also demonstrate that these drugs robustly decrease metastasis *in vivo* in nude mice. In addition, the treated mice did not show any alarming physical side effect at the end of the experiments (as assessed by weight measurements) other than drowsiness. Notably, it is hard to compare the doses we used in these experiments to those used in treated patients, especially since the mode of administration is different and the pharmacokinetics (blood concentrations, half-life and metabolism) of these compounds is unknown in our studies. However, because phenothiazines are FDA-approved and used in the clinic, their tolerability and toxicity levels in humans are already known. As such, the drowsiness side effect in mice remains a minimal issue to be addressed in future work. Indeed, further studies on TNBC pre-clinical models would complement our study before repurposing these drugs for TNBC patients with the goal of defining the most effective dosage in terms of diminishing tumor burden and metastatic incidences while minimizing the associated side effects.

Currently, advanced TNBC is mainly managed by chemotherapy. The lack of targeted therapies for this disease and the risk of developing resistance constitute bottlenecks in patient care and therapeutic strategies. AXL is a known mediator of resistance to different therapies including Receptor Tyrosine Kinase inhibitors, chemotherapeutics and radiation [5]. Therefore, the *AXL* gene signature identified here has the potential to reveal drugs that could sensitize TNBC cells to traditional therapies. Indeed, our work on TNBC cells and other studies in different cancer cell types have shown that phenothiazines can increase the sensitivity to traditional therapies including chemotherapy and radiotherapy [27, 34, 35]. Nonetheless, most of the studies, including our own, have been carried out using immunocompromised mice and it has been established that immune cells greatly modulate the efficacy of traditional therapies. Nevertheless, immunocompetent mouse cancer models were successfully treated with THZ in two different studies, suggesting that the effect on tumor growth is maintained in immunocompetent models [21, 24].

Altogether, we show that THZ, FLZ and TFP are potential candidates to be repurposed for the treatment of TNBC due to their ability to limit tumor growth and metastatic burden in mouse models. Metastasis being an undefeatable clinical obstacle, these drugs could be a new approach to test for improving the survival and quality of life of TNBC patients. In conclusion, our pharmacological approach, using an *AXL* gene signature, facilitated the identification of approved and clinically available drugs for repurposing in the treatment of TNBC. Thus, the identified compounds hold potential for TNBC treatment and constitute good candidates for further clinical testing in combination with current chemotherapeutic approaches.

## MATERIALS AND METHODS

### Cell culture and treatments

Human breast cancer cells MDA-MB-231-Luc [36] and Hs578T were maintained in DMEM with 10% FBS supplemented with ZellShield (Minerva). Cells were treated with Thioridazine (Sigma), Fluphenazine (Sigma), Trifluoperazine (Sigma), R428 (Apexbio) and paclitaxel (Sigma) at the indicated concentrations and time. Phenothiazines were resuspended in water and R428 and paclitaxel in DMSO. For experiments including siRNA, cells were transfected with a control siRNA or an ON-TARGET Smart Pool siRNA specific to AXL (Dharmacon) using Lipofectamine 2000 (Thermo Fisher Scientific).

### RNA sequencing and pharmacogenomics analyses

Total RNA was extracted from cells of the indicated treatments using the RNeasy column kit (QIAGEN). Deep sequencing was performed using the Illumina HiSeq 2000 platform at the Génome Québec Innovation Centre (McGill University). The differential expression measurements were performed with DESeq2 v1.4.5. GEO accession number: GSE120268. Gene ontology analyses were conducted using Gene Ontology Consortium [37] and GSEA v2.1.0 (Gene Set Enrichment Analysis) [17, 38]. For pharmacogenomics analysis, we used the PharmacoGx platform (version 1.1.5) to leverage the Connectivity Map data for drug repurposing analyses [14].

### Invasion assay

Boyden cell invasion assays were performed using 8 μm pore Boyden Chambers (Costar) with the upper chamber coated with 6 mL of Matrigel (BD Biosciences). 50,000 cells were seeded in the top chamber and allowed to invade for 16h toward the bottom chamber containing 10% FBS in DMEM. Cells were then fixed with 4% PFA and stained using SlowFade Gold reagent (Invitrogen) before counting the cells that migrated to the underside of the membrane using Leica DM6 microscope. Each experiment was performed in triplicate.

### Cell migration analysis by time-lapse microscopy

Cell migration tracking experiments were conducted as described in reference [10]. For most experiments, 24 h prior to imaging, cells were plated in 12 well plates and drugs were added 1h before imaging. For cell migration tracking on collagen, cells were plated in serum-free media in 12 well plates coated with collagen (5 μg/cm^2^) 2 h prior imaging cells. Drugs were added 1h before imaging. To generate time-lapse movies, pictures were taken every 10 minutes for 6h with a DM IRE2 microscope (Leica) equipped with an automated stage and controlled environment (PECON). Orca-ER Model C-4742 digital camera (Hamamatsu) was used and migration speed data and plots were generated with Volocity software (PerkinElmer Life and Analytical Sciences). For collagen plated experiments, quantification was done manually using Fiji. Briefly, 90 healthy cells per conditions were tracked manually for at least 20 pictures and average speed was used to compare conditions.

### Proliferation and cell viability assays

MDA-MB-231 expressing Luciferase were plated in 24 well plates and left to adhere for 24 h. The next day, the indicated concentration of drugs were added to the wells and Beetle Luciferin (Promega) was used to quantify the cells using the Xenogen IVIS 200 with Living Image 4.2 software (PerkinElmer). For proliferation, the plates were imaged after 24 h, 48 h and 72 h of treatment and for the viability assay, the plates were imaged after 24 h.

### Flow cytometry analysis

For cell cycle analysis, cells were treated as indicated in the figures for 24 h. BrdU (BD Pharmingen) was incorporated in cells for 2 h and cells were then fixed with EtOH. Denaturation was then done using 2N HCl that was neutralised with Na_2_B_4_O_7_. Anti-BrdU (BD Pharmingen) was then incubated 1h followed by a secondary antibody Alexa-Fluor 350 (Life Technologies) and counterstained with Propidium Iodide (PI) (Sigma-Aldrich). For apoptosis analysis, cells were stained using Annexin V-FITC (Abcam) and counterstained with PI. All FACS acquisitions and analysis were done using BD LSR Fortessa and FACSDiva software (BD).

### Tumorsphere formation assay

MDA-MB-231 and Hs578T cells were plated into low adherence plates in DMEM/F12 media supplemented with 0.4% FBS, EGF (20 ng/mL), FGF (10 ng/mL), Insulin (5 μg/mL) and B27 supplements (Invitrogen 17504-044) as described in [39]. Briefly, compounds were added at time of the plating in the tumorsphere media at indicated concentration and kept during all the experiment. 7 days later, the number of tumorspheres was determined manually using a DM IRE2 microscope (Leica).

### Western blot analyses

Cells were treated with the indicated drugs for 1 h before they were lysed in NP-40 buffer (150 mM NaCl, 50 mM Tris pH 7.5, 1% Nonidet P-40) supplemented with complete protease (Roche) and phosphatase inhibitors (Sodium Orthovanadate 1 μM, NaF 0,5 M). Proteins were quantified using the DC Protein Assay Kit (Bio-Rad) and a total of 80 μg of protein lysate was separated by SDS/PAGE to be detected by immunoblotting using Li-Cor technology.

### Antibodies

FACS: Anti-BrdU (1:200, BD Pharmingen). Western Blot: mTOR (1:1000, Cell Signaling), p-mTOR (1:1000, Cell signaling), AKT (1:1000, Cell signaling), p-AKT Ser473 (1:1000, Cell Signaling), ERK1/2 (1:1000, Cell Signaling), p-ERK Thr202/Tyr204 (1:1000, Cell Signaling), AXL (1:10 000, Genscript), p-AXL Tyr702 (1:1000, Cell Signaling) and Tubulin (1:10 000, Sigma).

Secondary antibodies: goat anti-rabbit IgG (IRDye 680RD, 1:10 000) and goat anti-mouse IgG (IRDye 800CW, 1:10 000) were purchased from Li-Cor.

### Animal experiments

A total of 32 Nude female mice were purchased from Jackson Laboratories and used at 3 weeks of age. Mice were housed in a specific pathogen-free (SPF) facility and experiments were approved by the Animal Care Committee of the Institut de Recherches Cliniques de Montréal (IRCM) and complied with the Canadian Council of Animal Care guidelines. For the experiments 4 groups of 8 mice were made randomly.

### Mammary fat pad grafts experiments and bioluminescence imaging

Mammary fat pad xenografts in Nude mice and bioluminescence imaging were previously described [10]. Briefly, a total of 10^6^ MDA-MB-231-Luc cells were injected in the cleared mammary fat pad of Nude mice of 3 weeks of age. The treatments began 10 days after the graft. Thioridazine (25 mg/kg), Fluphenazine (40 mg/kg), Trifluoperazine (25 mg/kg) and paclitaxel (10 mg/kg) were injected intraperitoneally 3 times a week for 4 weeks. Phenothiazines were administered in a PBS solution and paclitaxel was administered in corn oil. Tumor diameter was measured before the beginning of the treatment (10 days after the graft) to quantify the fold increase overtime. Randomisation was performed for injection and treatments.

To detect lung metastasis, 300 mg/kg of Beetle Luciferin (Promega) solution was injected intraperitoneally 10 minutes before harvesting the lungs for imaging. Bioluminescence imaging was done using a Xenogen IVIS 200 (PerkinElmer) and the Living Image 4.2 software. To measure bioluminescence, radiance was calculated for each lung using a circular region of interest.

### Immunohistochemistry (IHC) and TUNEL assay

Paraffin embedded tumors were cut at 5 μm and deparaffinized in xylene then rehydrated with an ethanol gradient. An antigen retrieval protocol was applied using 10mM Sodium citrate (pH 6) prior to blocking the sections in 3% H_2_O_2_. Sections were permeabilized with PBSTT (1X PBS, 0,5% Triton-X100, 0,02% Tween) and slides were further blocked in PBSTT containing 1% BSA. The sections were consequently incubated with a primary antibody against Ki67 (1:250, Medicorp), a biotin-conjugated anti-rabbit IgG secondary antibody (1:1000, BA-1000 Vector Laboratories) and a Streptavidin-HRP ternary antibody (1:1000, BD Pharmingen). DAB detection kit (Vector Laboratories) was then used to reveal the staining and a counterstain using Mayer’s Hematoxylin solution (Sigma) was performed. Apoptosis detection was performed following DeadEnd Fluorometric TUNEL system (Promega) and Hoechst was used to counterstain the nuclei (Thermo Fisher Scientific).

### Statistics

Data are presented as mean ± SEM from 3 or more independent experiments. Statistical analyses were performed using GraphPad Prism Software and the Student’s *t* test (comparison of two independent groups). *P*-values < 0.05 were considered as significant (^*^p < 0.05, ^**^p < 0.001, ^***^p < 0.0001).

## SUPPLEMENTARY MATERIALS FIGURES, TABLE AND VIDEO







## Abbreviations

EMT: Epithelial to Mesenchymal Transition
FLZ: Fluphenazine
TNBC: Triple Negative Breast Cancer
THZ: Thioridazine
TFP: Trifluoperazine
