# Supplementary material for: AXL knockdown gene signature reveals a drug repurposing opportunity for a class of antipsychotics to reduce growth and metastasis of triple-negative breast cancer
Source: Oncotarget. 2019 Mar 12;10(21):2055–67. doi: 10.18632/oncotarget.26725 (PMC6459349; doi:10.18632/oncotarget.26725)
Supplement: Supplementary file 1 [file oncotarget-10-2055-s001.pdf]

AXL knockdown gene signature reveals a drug repurposing opportunity for a class of antipsychotics to reduce growth and metastasis of triple negative breast cancer

SUPPLEMENTARY MATERIALS

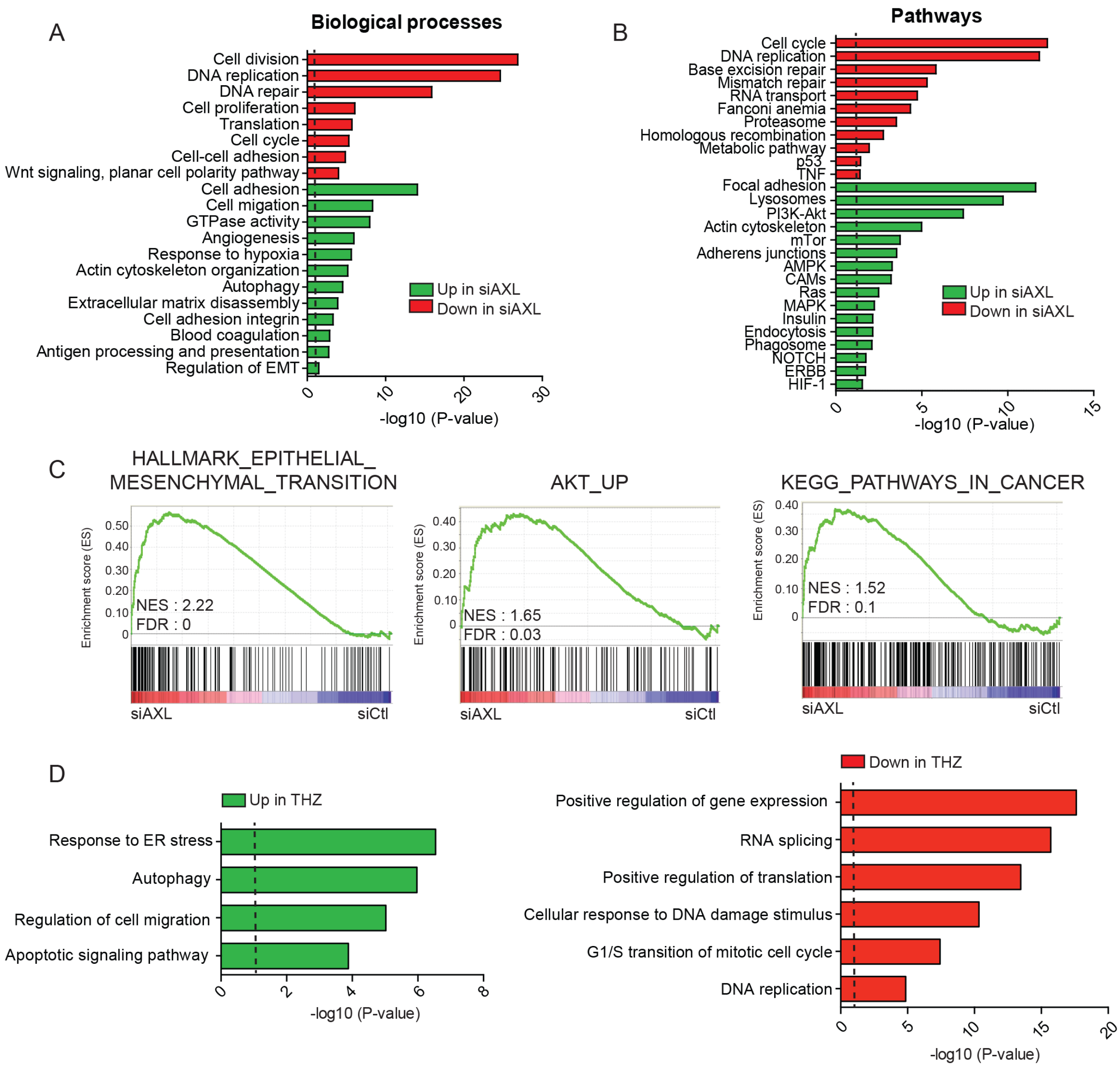

Supplementary Figure 1: Gene expression signature of AXL knockdown in MDA-MB-231 reflects AXL roles in different biological processes and oncogenic pathways. (**A–B**) Gene Ontology of MDA-MB-231 siAXL gene signature showing modulated biological processes and pathways. (**C**) GSEA analysis of the AXL depletion gene signature presents a role for AXL in EMT, AKT signaling and other general pathways implicated in cancer. (**D**) Gene Ontology of MDA-MB-231 treated with 10 μM THZ for 6 h present similar features found in siAXL signature.

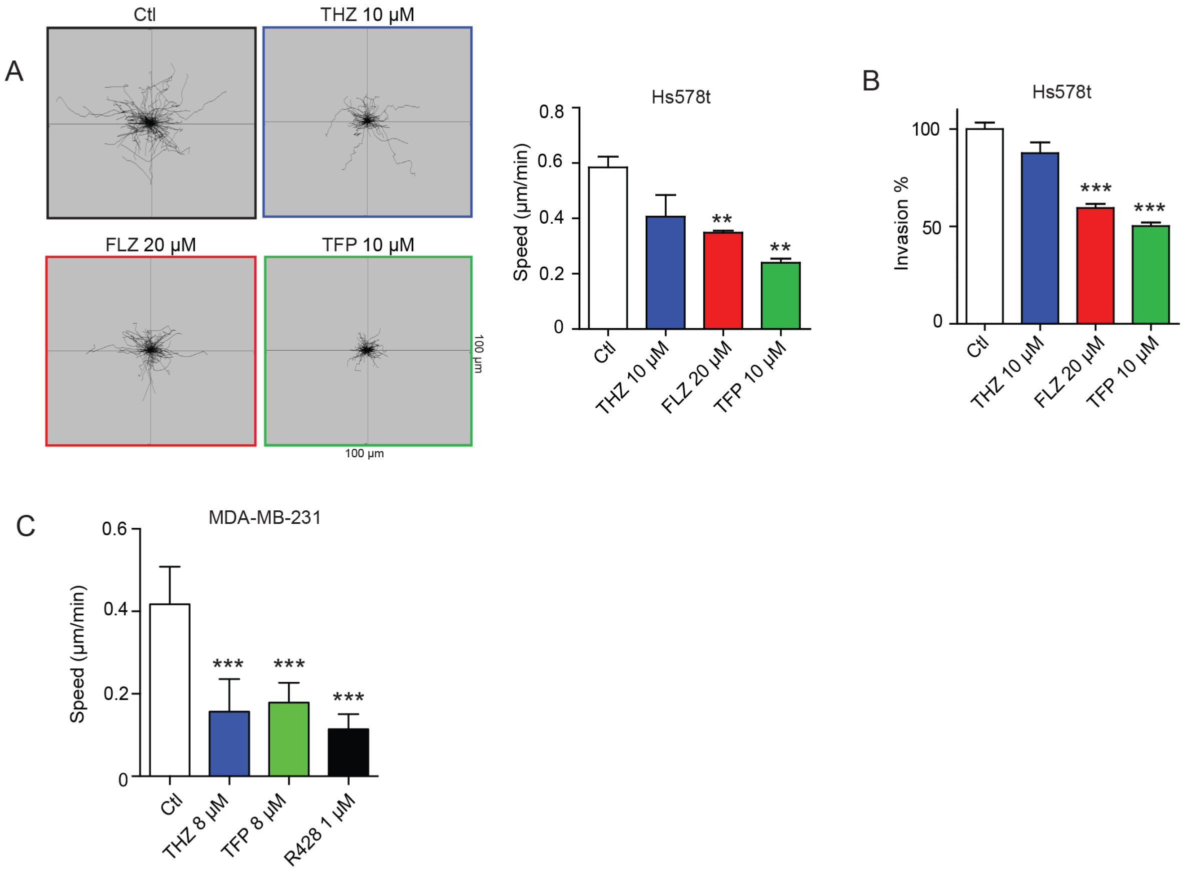

Supplementary Figure 2: Treatment with THZ, FLZ and TFP reduces the migration ability of Hs578t TNBC cells. (**A**) Time-lapse experiments over a 6 h period in the presence of phenothiazines show that these treatments reduce the migration speed of Hs578t TNBC cells ($^{**}p = 0.0039$, $^{**}p = 0.0012$). Data are represented as mean ± SEM. (**B**) Treatment with the antipsychotics reduces invasion of Hs578t in a Boyden invasion assay toward FBS ($^{***}p < 0.0001$). Data are represented as mean ± SEM. (**C**) Time-lapse experiments on MDA-MB-231 were performed on collagen coated plates confirming that phenothiazines and R428 reduce migration speed. FLZ wasn't included since it induced cell death in these conditions.

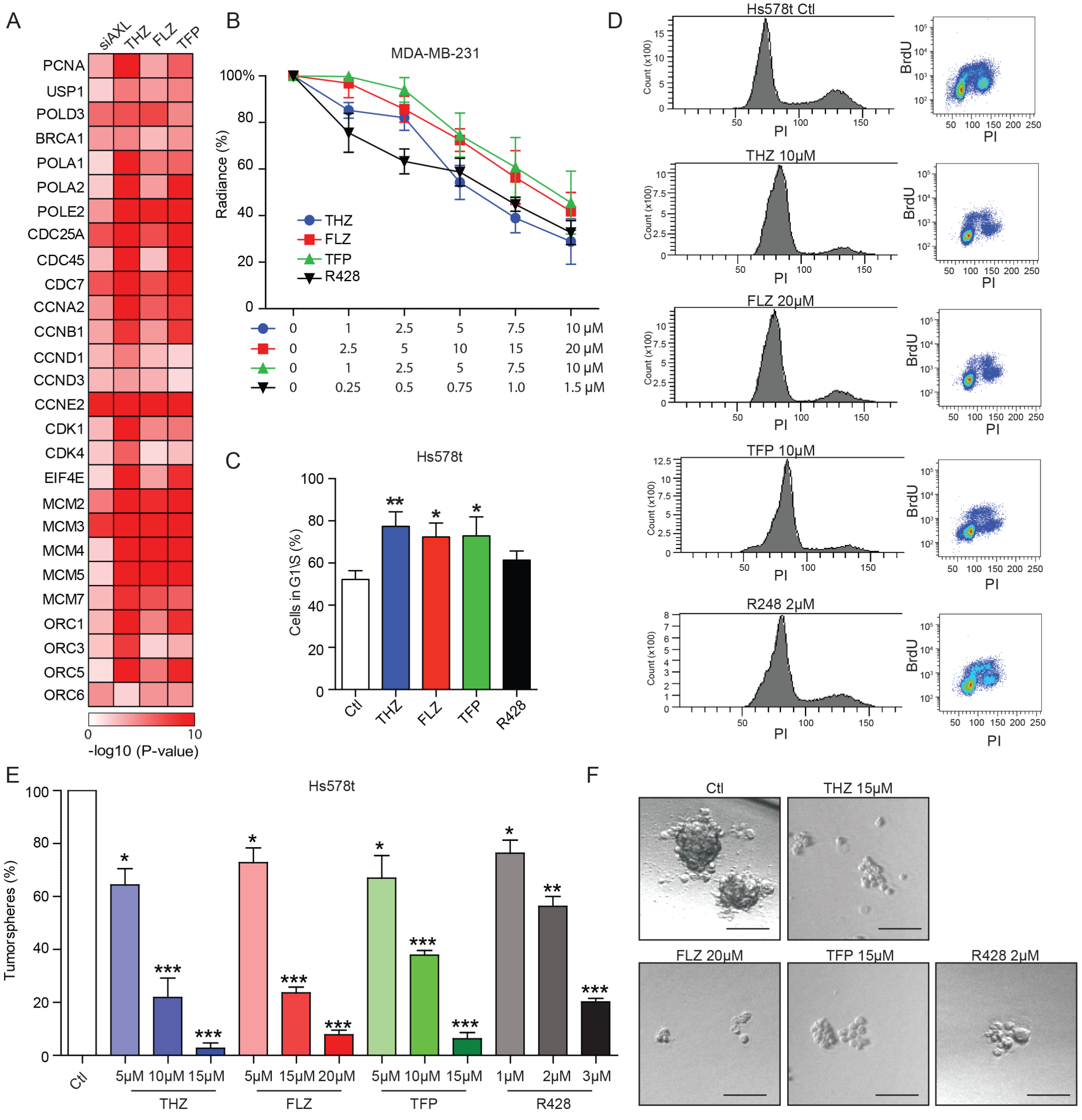

Supplementary Figure 3: THZ, FLZ and TFP induce cell cycle arrest and reduce cell proliferation of TNBC cells. (**A**) Heatmap representing the downregulation of genes (*p*-value) related to cell cycle and G1/S transition in cells treated with siAXL, THZ, FLZ and TFP. (**B**) The antipsychotics and AXL inhibitor R428 reduce the proliferation of MDA-MB-231-Luc cells in a dose dependant manner as shown by cell quantification using luminescence (radiance) after 72 h treatment at indicated concentrations ($n = 4$). (**C**–**D**) Treatment with the phenothiazines induces a cell cycle arrest in G1/S. Cell cycle analysis of Hs578t was performed using a BrDU staining assay by flow cytometry ($^{**}p = 0.0059$, $^{*}p = 0.0110$, $^{*}p = 0.0229$) ($n = 3$). Data are represented as mean ± SEM. (**E**–**F**) Tumorspheres formation of Hs578t is inhibited by the treatment with THZ, FLZ, TFP and R428 in a dose-dependant manner ($^{*}p = 0.0184$, $^{***}p = 0.0009$, $^{***}p < 0.0001$, $^{*}p = 0.0416$, $^{***}p < 0.0001$, $^{*}p = 0.0307$, $^{***}p = 0.0002$, $^{***}p < 0.0001$, $^{*}p = 0.0013$, $^{**}p = 0.0029$, $^{***}p < 0.0001$) ($n = 3$). Scale bar, 150 µm. Data are represented as mean ± SEM.

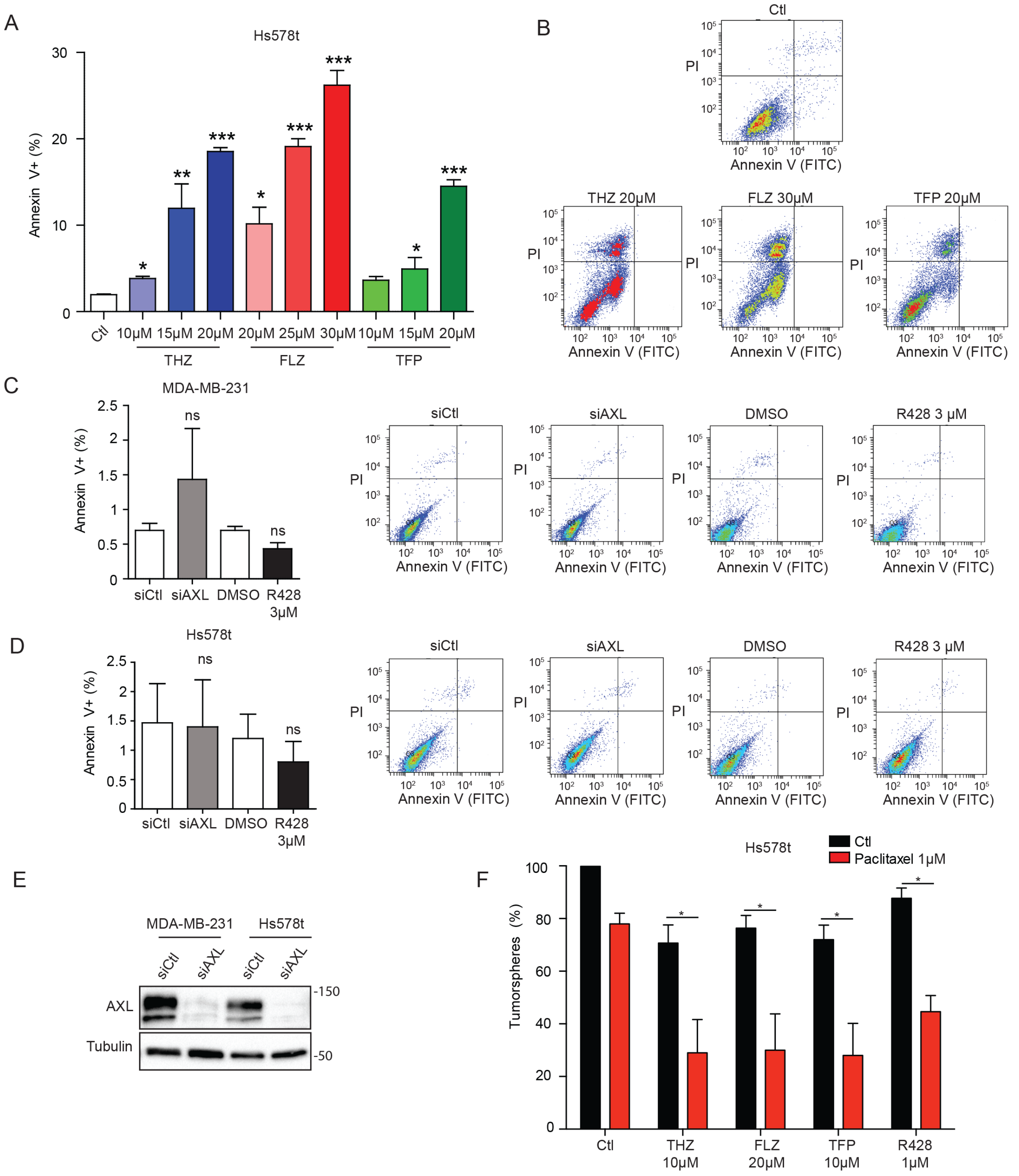

Supplementary Figure 4: THZ, FLZ and TFP exhibit cytotoxic effects on Hs578t. (**A–B**) Treatment with the phenothiazines during 24 h at indicated concentration induces the apoptosis of Hs578t cells. Flow cytometry analyses of Annexin V staining was used. ($^{*}p = 0.0155$, $^{*}p = 0.0254$, $^{**}p = 0.0026$, $^{*}p = 0.0142$, $^{***}p < 0.0001$, $^{*}p = 0.0203$, $^{***}p < 0.0001$). Data are represented as mean ± SEM. (**C–D**) AXL inhibition or AXL knockdown does not induce apoptosis of MDA-MB-231 or Hs578t TNBC cells in an Annexin V assay. ($n = 3$) Data are represented as mean ± SEM. (**E**) Validation of AXL knockdown by siRNA by western blot. (**F**) Combination of paclitaxel and the phenothiazines or R428 increase the effect of these drugs on tumorspheres formation of Hs578t ($^{*}p = 0.0211$, $^{*}p = 0.0289$, $^{*}p = 0.0175$, $^{*}p = 0.0102$). ($n = 3$) Data are represented as mean ± SEM.

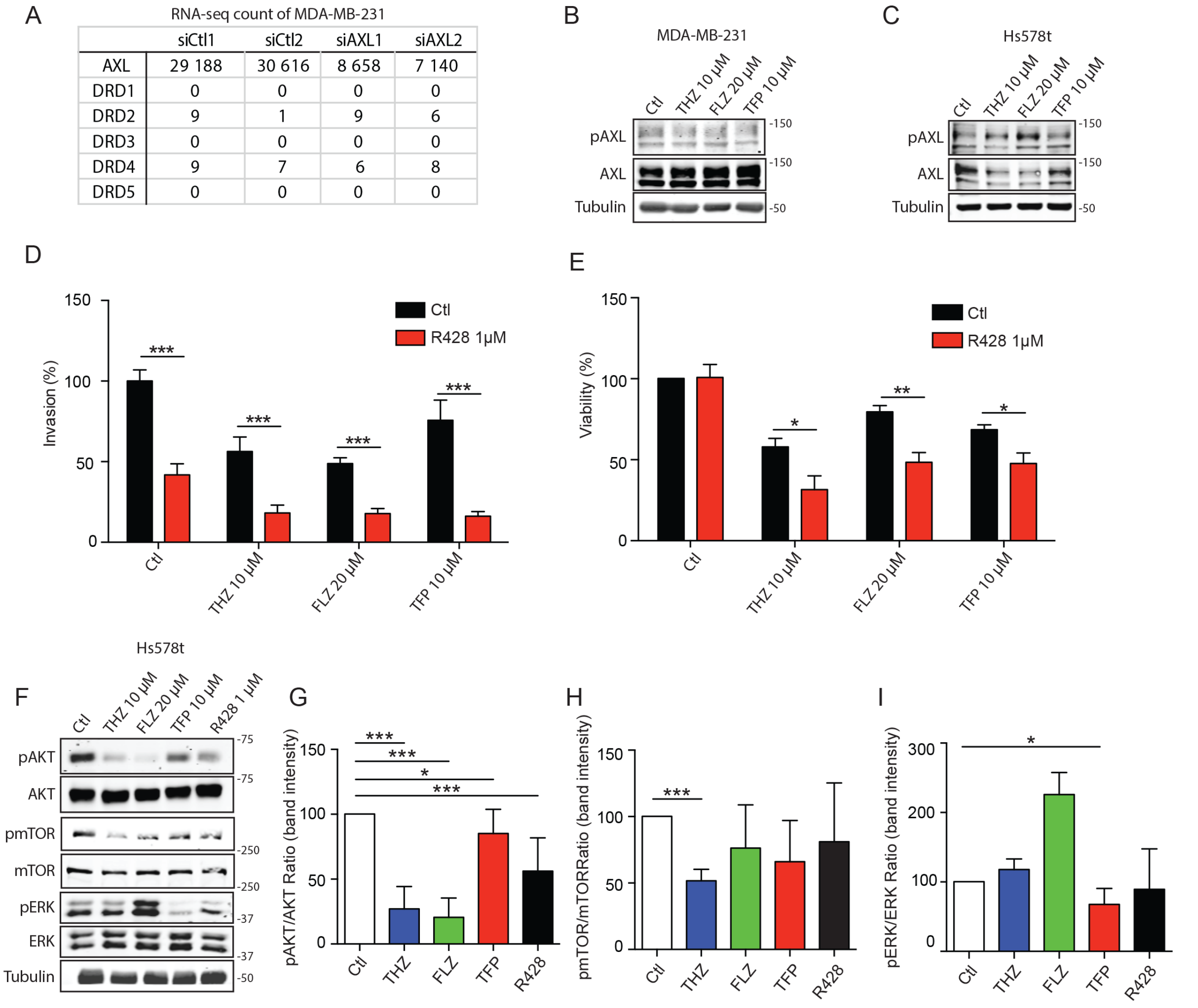

Supplementary Figure 5: THZ, FLZ and TFP treatment affect PI3K/Akt/mTOR without affecting AXL activity. (**A**) RNA-seq count of MDA-MB-231 showing that these cells do not express any dopamine receptors. (**B–C**) Treatment with THZ, FLZ or TFP does not affect AXL phosphorylation in MDA-MB-231 and Hs578t. (**D–E**) Combination of the antipsychotics and AXL inhibitor R428 increases the effects of these drugs on cell invasion and cell viability ($^{***}p < 0.0001$/$^{*}p = 0.0456$, $^{**}p = 0.0047$, $^{*}p = 0.0283$). ($n = 3$) Data are represented as mean ± SEM. (**F**) Treatment with phenothiazines and R428 modulates the phosphorylation of AKT, mTOR and ERK in Hs578t. (**G–I**) Quantification of this decrease using a ratio of band intensity of the phosphorylated form over the total protein.

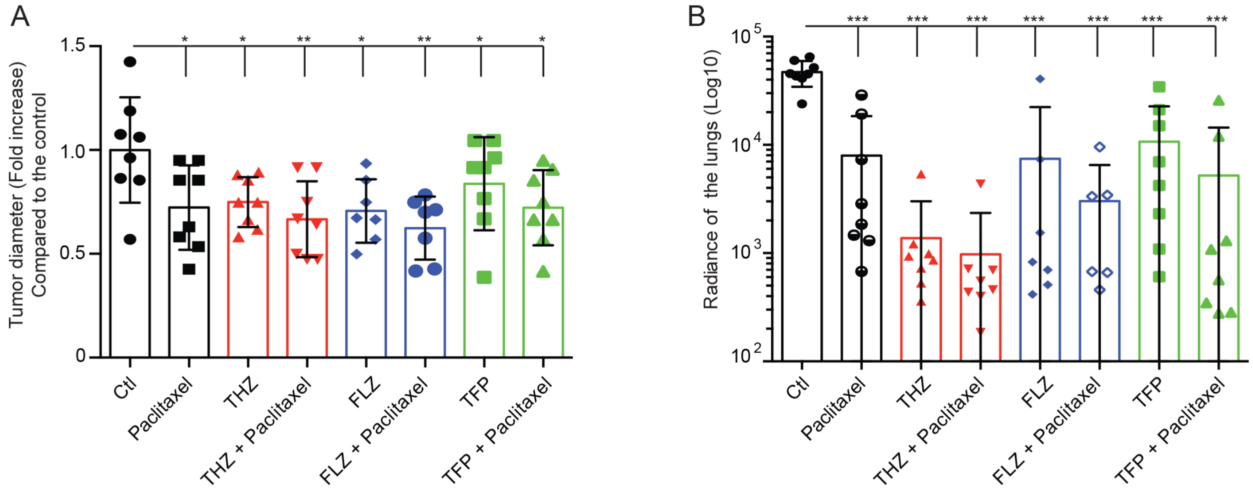

Supplementary Figure 6: Combination of THZ, FLZ or TFP with paclitaxel reduces tumor and metastatic burden *in vivo*. (**A**) Treatment of MDA-MB-231-Luc fat pad grafts with the phenothiazines and paclitaxel reduces tumor growth. Tumor diameter was measured before the beginning of the treatment (7 days after the graft) to quantify the fold increase overtime. Experiment was stopped at 31 days after the graft ($^{*}p = 0.0304$, $^{*}p = 0.0242$, $^{**}p = 0.0093$, $^{*}p = 0.0197$, $^{**}p = 0.0046$, $^{*}p = 0.0344$, $^{*}p = 0.0244$). ($n = 6–8$) Data are represented as mean ± SEM. The conditions Ctl, THZ, FLZ and TFP are the same as in Figure 6C. (**B**) Treatment with the antipsychotics and paclitaxel reduces the metastatic progression of MDA-MB-231-Luc xenografts to the lungs. Lungs were dissected and imaged for bioluminescence signal 31 days after tumor engraftment, at the end of the growth experiment. ($^{***}p < 0.0001$) Data are represented as mean ± SEM. The conditions Ctl, THZ, FLZ and TFP are the same as in Figure 6C.

Supplementary Table 1 : Connectivity analysis of drugs with a similar gene signature to AXL decrease. See Supplementary_ Table_1

Supplementary Movie 1: Video time lapse microscopy of MDA-MB-231 cells treated with vehicle (Control) or THZ, FLZ or TFP drugs. Images were acquired every 10 minutes for 6 hours and were used to generate the movies. See Supplementary_ Movie_1
