# Supplementary material for: AXL knockdown gene signature reveals a drug repurposing opportunity for a class of antipsychotics to reduce growth and metastasis of triple-negative breast cancer
Source: Oncotarget. 2019 Mar 12;10(21):2055–67. doi: 10.18632/oncotarget.26725 (PMC6459349; doi:10.18632/oncotarget.26725)
Supplement: Supplementary file 2 [file oncotarget-10-2055-s002.docx]

Supplementary Table 1 : Connectivity analysis of drugs with a similar gene signature to AXL decrease

| Compound | Connectivity | P Value |
| --- | --- | --- |
| STOCK1N-35696 | 0.760215 | 0.048786099 |
| trifluoperazine | 0.48112 | 0.000892695 |
| thioridazine | 0.466335 | 0.000363537 |
| sirolimus | 0.465605 | 0.000936876 |
| fluphenazine | 0.462585 | 5.73E-05 |
| prochlorperazine | 0.4583 | 0.000471357 |
| LY-294002 | 0.457575 | 9.68E-05 |
| 5109870 | 0.44616 | 0.000147726 |
| 0297417-0002B | 0.443045 | 8.05E-05 |
| wortmannin | 0.42249 | 0.000104494 |
| nortriptyline | 0.416855 | 0.000112603 |
| ciclopirox | 0.415545 | 0.000110677 |
| chlorpromazine | 0.410835 | 0.000105068 |
| levomepromazine | 0.40536 | 5.94E-05 |
| cloperastine | 0.403275 | 8.04E-05 |
| tonzonium bromide | 0.402465 | 0.000105283 |
| methylbenzethonium chloride | 0.399725 | 0.000100575 |
| norcyclobenzaprine | 0.39664 | 0.000103414 |
| protriptyline | 0.395965 | 5.64E-05 |
| syrosingopine | 0.394145 | 5.50E-05 |
| fulvestrant | 0.39319 | 5.31E-05 |
| promazine | 0.39039 | 5.52E-05 |
| CP-645525-01 | 0.386385 | 0.000540382 |
| resveratrol | 0.38461 | 7.05E-05 |
| 0175029-0000 | 0.383935 | 0.001415278 |
| alsterpaullone | 0.383425 | 0.003168117 |
| quinostatin | 0.38237 | 0.007928474 |
| phenazopyridine | 0.382245 | 7.09E-05 |
| metergoline | 0.382065 | 7.14E-05 |
| geldanamycin | 0.38005 | 6.77E-05 |
| perhexiline | 0.378515 | 0.000126951 |
| monensin | 0.376525 | 6.46E-05 |
| loperamide | 0.375515 | 7.13E-05 |
| trimipramine | 0.3723 | 0.000575889 |
| PF-00539758-00 | 0.37053 | 5.20E-05 |
| metixene | 0.368635 | 5.78E-05 |
| homochlorcyclizine | 0.368075 | 5.62E-05 |
| rifabutin | 0.36648 | 5.62E-05 |
| erastin | 0.36488 | 5.20E-05 |
| perphenazine | 0.36435 | 0.000104275 |
| maprotiline | 0.363655 | 7.55E-05 |
| doxorubicin | 0.361145 | 0.007900847 |
| raloxifene | 0.35951 | 0.000428642 |
| famprofazone | 0.35946 | 9.29E-05 |
| etacrynic acid | 0.357435 | 0.000198679 |
| tanespimycin | 0.356705 | 8.80E-05 |
| irinotecan | 0.35417 | 0.001280348 |
| astemizole | 0.35359 | 5.97E-05 |
| HC toxin | 0.3514 | 6.15E-05 |
| co-dergocrine mesilate | 0.35139 | 0.000118941 |
| pimozide | 0.351165 | 6.18E-05 |
| fluspirilene | 0.348785 | 9.87E-05 |
| vorinostat | 0.348725 | 5.58E-05 |
| GW-8510 | 0.34816 | 7.24E-05 |
| camptothecin | 0.34815 | 0.007907755 |
| tetryzoline | 0.34526 | 5.81E-05 |
| pyrvinium | 0.344625 | 9.49E-05 |
| valproic acid | 0.34459 | 5.85E-05 |
| etoposide | 0.343635 | 5.33E-05 |
| ellipticine | 0.343555 | 0.000220742 |
| withaferin A | 0.34254 | 5.18E-05 |
| 0173570-0000 | 0.34175 | 5.28E-05 |
| benzethonium chloride | 0.34174 | 8.59E-05 |
| scriptaid | 0.34114 | 0.001004933 |
| H-7 | 0.341135 | 0.000274399 |
| bromocriptine | 0.339505 | 0.000232433 |
| AG-013608 | 0.334985 | 9.56E-05 |
| alvespimycin | 0.333335 | 5.36E-05 |
| thioguanosine | 0.333255 | 0.001028491 |
| Prestwick-559 | 0.333 | 0.000100309 |
| dihydroergocristine | 0.332485 | 6.83E-05 |
| benzamil | 0.3316 | 0.00013243 |
| clomifene | 0.330385 | 0.000380431 |
| mepacrine | 0.33008 | 0.000132255 |
| phenoxybenzamine | 0.32954 | 5.66E-05 |
| chlorcyclizine | 0.32794 | 6.13E-05 |
| niclosamide | 0.327735 | 5.55E-05 |
| sulfapyridine | 0.32553 | 6.18E-05 |
| amoxapine | 0.32404 | 0.000227628 |
| clomipramine | 0.323895 | 0.000715475 |
| colforsin | 0.323715 | 0.000210578 |
| pergolide | 0.323585 | 7.13E-05 |
| methotrexate | 0.322645 | 5.35E-05 |
| amiodarone | 0.322575 | 0.000168514 |
| thiethylperazine | 0.32239 | 7.09E-05 |
| fluoxetine | 0.3222 | 5.70E-05 |
| deptropine | 0.320985 | 0.000100538 |
| saquinavir | 0.32042 | 5.93E-05 |
| ciclosporin | 0.32038 | 0.00014725 |
| gossypol | 0.320115 | 6.16E-05 |
| clozapine | 0.31913 | 0.000299923 |
| azacitidine | 0.319125 | 0.004308051 |
| rescinnamine | 0.318965 | 9.85E-05 |
| parthenolide | 0.318535 | 5.66E-05 |
| proscillaridin | 0.31794 | 0.000495004 |
| staurosporine | 0.316645 | 5.27E-05 |
| terfenadine | 0.315935 | 6.36E-05 |
| lanatoside C | 0.31501 | 0.001127296 |
| CP-944629 | 0.314365 | 0.000116165 |
| trichostatin A | 0.314245 | 5.79E-05 |
| promethazine | 0.314185 | 5.27E-05 |
| piperacetazine | 0.31406 | 5.49E-05 |
| mebhydrolin | 0.31363 | 0.000182508 |
| dihydroergotamine | 0.313445 | 7.51E-05 |
| mefloquine | 0.31333 | 0.000102549 |
| propafenone | 0.313 | 0.007900847 |
| mitoxantrone | 0.31153 | 0.001035022 |
| flupentixol | 0.310585 | 8.01E-05 |
| alexidine | 0.3098 | 0.000270726 |
| mebendazole | 0.30936 | 7.87E-05 |
| cyproheptadine | 0.30861 | 5.26E-05 |
| trifluridine | 0.308515 | 6.67E-05 |
| fluvoxamine | 0.308385 | 5.60E-05 |
| sulconazole | 0.307645 | 0.000100656 |
| lovastatin | 0.307585 | 8.53E-05 |
| fluvastatin | 0.30716 | 7.63E-05 |
| alimemazine | 0.306465 | 0.002342371 |
| thioproperazine | 0.304345 | 0.000139439 |
| dipyridamole | 0.304325 | 0.000342891 |
| bufexamac | 0.30412 | 5.24E-05 |
| monorden | 0.30385 | 5.59E-05 |
| daunorubicin | 0.303035 | 0.000513021 |
| quinisocaine | 0.30198 | 0.000125182 |
| monobenzone | 0.301815 | 6.37E-05 |
| corbadrine | 0.30173 | 9.23E-05 |
| butoconazole | 0.30146 | 6.99E-05 |
| PNU-0230031 | 0.300975 | 0.000931543 |
| colchicine | 0.30063 | 5.99E-05 |
| tyrphostin AG-1478 | 0.300325 | 6.14E-05 |
| 15-delta prostaglandin J2 | 0.30023 | 0.000327755 |
| CP-319743 | 0.300045 | 6.58E-05 |
| ethaverine | 0.300015 | 5.82E-05 |
